# Composite Polymers from Leather Waste to Produce Smart Fertilizers

**DOI:** 10.3390/polym13244351

**Published:** 2021-12-12

**Authors:** Daniela Simina Stefan, Magdalena Bosomoiu, Rodica Roxana Constantinescu, Madalina Ignat

**Affiliations:** 1 Department of Analytical Chemistry and Environmental Engineering, Faculty of Applied Chemistry and Materials Science, University Politehnica of Bucharest, 1-7 Polizu Street, 011061 Bucharest, Romania; daniela.stefan@upb.ro; 2 Leather and Footwear Research Institute (ICPI) Division, National Research & Development Institute for Textiles and Leather, 93 Ion Minulescu Street, 031215 Bucharest, Romania; rodica.constantinescu@icpi.ro (R.R.C.); madalina.ignat@icpi.ro (M.I.)

**Keywords:** bio-polymer, hide waste, circular economy

## Abstract

The leather industry is facing important environmental issues related to waste disposal. The waste generated during the tanning process is an important resource of protein (mainly collagen) which can be extracted and reused in different applications (e.g., medical, agricultural, leather industry). On the other side, the utilization of chemical fertilizers must be decreased because of the negative effects associated to an extensive use of conventional chemical fertilizers. This review presents current research trends, challenges and future perspectives with respect to the use of hide waste to produce composite polymers that are further transformed in smart fertilizers. Hide waste contains mostly protein (collagen that is a natural polymer), that is extracted to be used in the cross-linking with water soluble copolymers to obtain the hydrogels which are further valorised as smart fertilizers. Smart fertilizers are a new class of fertilizers which allow the controlled release of the nutrients in synchronization with the plant’s demands. Characteristics of hide and leather wastes are pointed out. The fabrication methods of smart fertilizers and the mechanisms for the nutrients release are extensively discussed. This novel method is in agreement with the circular economy concepts and solves, on one side, the problem of hide waste disposal, and on the other side produces smart fertilizers that can successfully replace conventional chemical fertilizers.

## 1. Introduction

It has been demonstrated that crop quality and yield is closely related to the type and concentration and release mode of fertilizers used. Nitrogen, carbon and phosphorous are essential nutrients for the growth of plants. Over the years, it has been evidenced that most of the chemical synthetic fertilizers have reduced efficiency in time, because of volatilization, leaching due to their good mobility all together with the disadvantage of necessity to apply large quantities frequently. This creates environmentally related problems regarding water, air and soil pollution, such as water contamination (especially when these substances penetrate below the plant’s roots and pollute the ground water), eutrophication, soil erosion, food contamination and effective hazardous emissions. Nitrate and phosphate leaching has also been reported, due to an excess of nutrients release, which are further transported from soils to water, causing the eutrophication [1,2,3]. Over long-term the extensive use of synthetic fertilizers could cause even a more reduced soil fertility, because of the increased need of food quantities. Another problem associated with inappropriate fertilization practices include low disease resistance of crops, causing a decrease of the productivity and poor-quality crops [4].

To reduce the environmental impact, chemical fertilizer substitutes, can be applied (e.g., organic fertilizers, biofertilizers). Work has been done in developing new formula fertilizers that allow slower and controlled nutrient release in accordance with the plant life cycle [5].

Organic fertilizers such as animal manure or sewage sludge are used to increase the soil fertility in nitrogen, carbon and phosphorous nutrients [4,6], but this brings a series of problems related to the risk of accumulation of heavy metals and organic pollutants (phthalate esters) [7,8]. That is why some countries (e.g., Switzerland) have prohibited the use of such fertilizers [9].

Biological fertilizers contain different types of microorganisms that convert the main nutrients from an inaccessible to an accessible form, during biological processes, and lead to the development of root systems and better seed germination [10]. The mechanisms involved are complex and depend on the microorganism type; it has been found that bacteria Pseudomonas and Azotobacter combined with organic manures (vermicompost and farm yard manure) enhanced plant growth and determinate early flowering for strawberry [11].

To overcome all the above mentioned disadvantages, a new class of fertilizers is needed, which allows the controlled release of the nutrients in synchronization with the plants demands. In turn will enhance the efficiency of fertilizers use and optimize the fertilizers application, thus reducing the costs associated to this operation [12]. This new type of fertilizers is called smart fertilizers [13]. 

Another alternative to conventional fertilizers, that has recently received great attention is constituted by the hydrogels, because of their properties of water/aqueous solutions absorption and retention, as well as their slow release of the nutrients together with the absorbed water, when the soil humidity decreases [14]. These properties are highly influenced by the concentration and pH of the aqueous solution, and temperature [15]. The precursor of hydrogel, called superabsorbent, can absorb large amounts of solutions containing the nutrients (e.g., urea); the nutrients release is controlled by the concentration gradient between the hydrogel and the environment around the hydrogel (soil), which corresponds to the plant demand in nutrients [16]. Hydrogels have been initially used in agriculture only as an alternative water resource, because of their capacity to absorb water [17]. Therefore, the use of hydrogels contributes also to a better management of water resources by reducing the irrigation frequency and preventing water loss through evaporation. 

There are many studies dedicated to developing synthetic hydrogels [18,19,20,21,22,23].

## 2. Characteristics of Hide and Leather Wastes

The problematic disposal of hide waste has received a particular interest because of the high quantities that are generated from leather industry and its negative impact on the environment. Hide and skins are by-products in the meat industry, and raw material in the leather industry [24,25]. Over the last 20 years, a continuously increasing number of raw hides and skins has been seen, passing from about 470 thousand tons in 1999 to 574 thousand tons in 2014 for heavy leather, respectively from 11,978 million square feet in 1999 to 14,540 million square feet in 2014, for light leather [26]. This means that the quantity of hide waste generated is also increasing. 

More than 99% of the world leather production comes from the processing of raw hides and skins from animals raised mainly for milk and/or meat production. The leather industry produces solid, liquid and gaseous phases waste. About 20% of the raw hide is transformed in finished leather, the rest being lost during the manufacturing process (20 kg of leather can be obtained from 100 kg of raw hide) [27,28]. The solid waste consists of hair, trimmings, flesh, keratin [29,30,31]. A detailed presentation of the steps in the tanning process is given by Sundar et al. (2011) [27].

During the tanning process, the so called “wet blue”, a stable and inert polynuclear chromium-collagen complex, is formed. The next step of the fabrication process is to equalize the thickness of “wet blue” and to cut the uneven parts. In this way, large amounts of material from shavings and trimming are produced, around 40% of product turned into waste by this stage [32].

**Figure 1:**
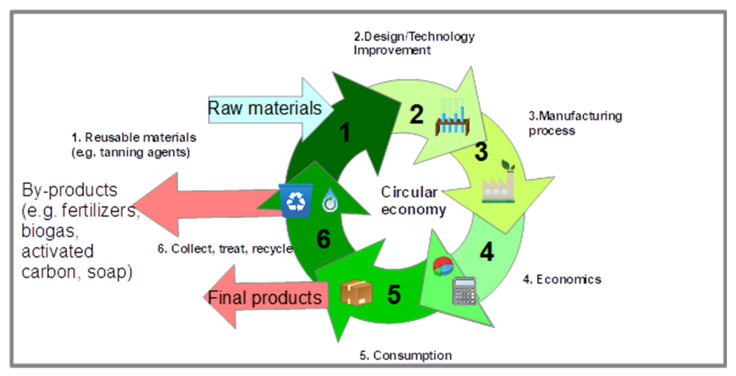
Circular economy in leather industry.

The circular economy concept has gathered substantial regional and worldwide interest. According to this concept, the materials and resources must be recovered and reintegrated in the system at the end of their life cycles, by optimizing their potential use (Figure 1). This is done by recycling, reusing, repairing, considering that any residual stream can be used either in the same process, or to make a new product. The major obstacles encountered in developing a circular economy for the leather industry are: (1) significant environmental and social impacts of waste leather landfilling operations; (2) continuous increase of production of leather-based products, and, implicitly, of hide waste quantity, especially in developing countries and; (3) availability of very few alternative disposal methods to waste landfill.

The influence of the tannery process on the environment can be measured in significant changes of parameters like chemical oxygen demand (COD), total dissolved solids (TDS), chlorides, sulphates and heavy metal pollution. The chemical substances discharged in the aquatic systems accumulate and generate polluted sediments and rivers salinization. Regarding the negative impact reduction on environment by tannery processes, there are two main directions: first is related to optimizing the process technologies to decrease the load of streams in toxic compounds, the second one consists in recovery, treatment and reuse of wastes generated during the tanning process [33]. Although a lot of progress has been made regarding chrome recovery in the tanning step, there is still more to achieve regarding the use of hide waste and development of a good strategy that can be applied at large scale. The leather industry can be a resource of by-products obtained by the recovery and use of different generated raw materials such as chromium, nutrients, collagen hydrolysate, fats, biogas and anaerobic digestate, which can be reused in other applications (e.g., in agriculture as fertilizers, for energy generation as biofuels, and in pharmacy and cosmetics) [34].

Another aspect to be taken into account regards the new indications of the European Commission on the reduction by 30% of the use of fertilizers from non-renewable resources. This can be accomplished by the valorisation of wastes that are suitable for production of smart fertilizers. The use of hide waste is a practical solution to recover valuable fertilizer components (namely proteins like collagen). This involves the construction of small installations for fertilizer production at the site of waste generation, which will solve both the problem of waste transport and sanitary hazards [35].

Kilic et al. (2018) [36] analysed the case of a tannery in Turkey, and provided recommendations for the process improvement related to energy consumption reduction, use of renewable energy resources, waste management and the reduction of water consumption.

Vidaurre Arbizu et al. (2021) [37] studied the case of a tannery located in Navarra (North of Spain) following the concepts of circular economy. This tannery was producing around 2 tons/day of leather shavings and leather dust, and 10 tons/day of discarded hair. Three different types of solid wastes (chromium free tanned shavings, chromium buffing dust, and discarded hair) were analysed, in an attempt to recycle them. Two directions were proposed for the reuse of the weekly tons of leftovers (both shavings and hair) produced by the tannery, instead of the usual composting in an external composting plant, or by landfilling. The first direction was to use the discarded material to obtain biomass for the company’s thermal production plant. For that, the calorific value of the discarded hair and shavings was estimated, to see if they are of competitive value in comparison with actual biomass products (e.g., wood pellets). The second approach involved the use of tannery-generated waste in the construction sector, as acoustic panels.

**Figure 2:**
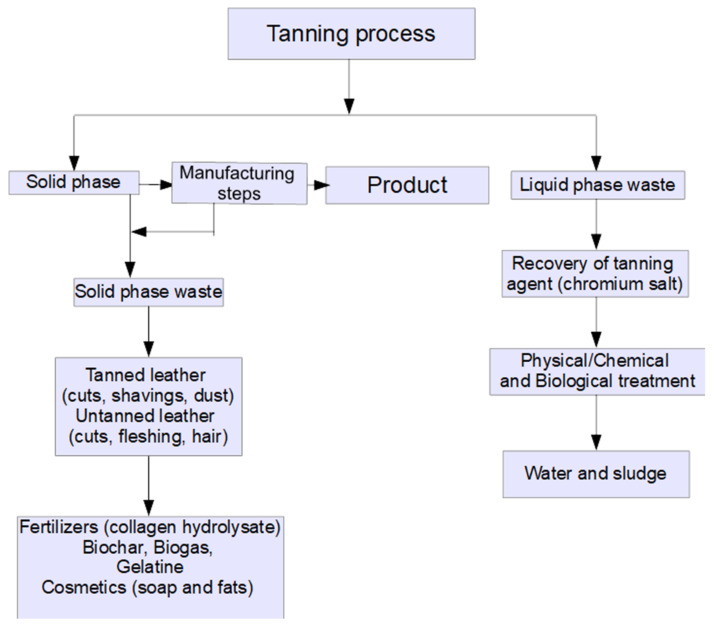
Tanning wastes and alternative ways for wastes valorisation.

Various uses or disposal methods have been reported for tannery waste, in the attempt to reduce its impact on the environment, and to create efficient models of circular economy in the leather industry [34,38]; among these methods there are: pyrolysis [39,40,41], biotransformation [42,43,44], use as adsorbent after transformation in activated carbon [28,45,46], biodiesel production [47], transformation into composite sheets [48] or doped nanocarbon [49], landfilling [50] etc. Alibardi and Cossu (2016) [50] proposed a sustainable method for landfilling of tannery sludge generated after the tannery wastewater treatment. The applied pretreatment processes consist of aerobic stabilization, compaction and drying, produced a reduction of volume, mass and biodegradability of treated sludge, demonstrating a reduced leachability of organic and inorganic compounds from the treated sludge. However, landfilling in the case of hide waste has numerous disadvantages related to the leaching of Cr (III) by the acid rains to the groundwater, soil contamination and high cost, on one side, and, on the other, that all the raw materials contained in the hide waste are not recovered (e.g., Cr (III) and proteins). Pyrolysis also presents a series of disadvantages regarding the gaseous emissions (HCH, NH_3_, as nitrogen is present in the form of amino acids in the leather), the ashes that contain Cr (III) or Cr (VI), depending on pH. It has been found that in the pH ranges 6.3–11.5, the dominant chromium species is Cr (III), whilst for pH above 11.5 the dominant species is Cr (VI) [51]. Torres-Filho et al. (2016) [52] studied the pyrolysis of leather wastes from tanning to obtain carbonized leather residue that is further used in metallurgical processes. Tang et al. (2021) [53] used non-tanned hide wastes to produce an efficient adsorbent for dye removal from tannery wastewater. In Figure 2, the possible utilizations of wastes generated during the tanning process are schematically presented.

One of the first steps in the tanning process is to remove the hairs from the hide, hairs that end up in the sludge, after the water treatment. Untanned skin waste can be transformed to produce organic derivatives, such as glue and gelatine [29]. Keratin hydrolysate and fleshing hydrolysate (after a chemical modification) can be used in the retanning process [29,54]. Fleshing wastes can also be used to produce glue, gelatine. Chrome and buffing dust are used to produce tanning agents, fertilizers etc. [29,55]. Fat and other tissues resulting from leather can be a source of biogas after an anaerobic treatment [56,57,58,59,60]. Puhazhselvan et al. (2017) [61] developed a method for the extraction in the presence of enzymes (Bacillus subtilis) of lipids from tannery fleshing waste, allowing the reducing of solvent consumption by 1.9 to 7 times corresponding to the production of 1 kg lipids, compared to conventional methods.

Pecha et al. (2021) [44] built a mathematical model based on experimental data obtained from enzymatic hydrolysis of chrome-tanned solid waste. This study includes experimental verification of reaction kinetics of alkali-enzymatic hydrolysis of chrome shavings and their dependence on enzyme concentration, time of reaction. The simulations showed the existence of a restraint range of optimal reaction conditions, corresponding to the minimum of unit operating costs.

Al-Jabari et al. (2021) [31] studied the case of a tannery implemented in Palestine, to better understand the possible directions to follow for a cleaner production, and to propose resource recovery technologies. To do that, the processes involved in the leather tannery have been reviewed, based on raw materials, processing parameters and effluent composition, changes being proposed at different stages (e.g., fleshing after liming, counter-current soak, use of new materials: enzymes for unhairing, and carbon dioxide and/or organic acid, for deliming and pickling, waste recycling: protein recycling from fleshing, salt collection and reuse, for hide preservation and waste recycling).

Smart fertilizers produced from hide waste protein hydrolysate (such as collagen) offer the advantages of valorising a large quantity of the wastes generated by the meat and leather industry. At the same time it determinates the obtaining of higher crop yields, with a lower cost, contributes to the conservation of the soil fertility (by not using the conventional chemical fertilizers) and combats environmental challenges related to waste disposal [62].

## 3. Hide and Leather Waste Processing

Sometimes, the hide waste needs to be processed for hair and flesh removal prior to its use for collagen recovery. The process uses lime and sodium sulphide to pulp the hair while the flesh is removed mechanically [49]. The processing of hides and skins involves multiple operations to achieve their conversion to the final products. Therefore, hide and skins trimming wastes are less contaminated by chemicals compared to the trimming generated by tanned and finished leathers. Tanning is the process by which the leather is given more stability and resistance to the chemical, thermal and biological degradation, by stabilizing the protein (collagen). This is done by chromium tanning which consists in the cross linking of collagen free carboxyl groups with the chromium ions [63]. It was found that around 15–30% of proteinous solid wastes generated from tanneries are chrome contaminated shavings, produced when the tanned hide is shaved to a uniform thickness [29,55]. As reported by Tahiri et al. (2007) [64], the chromium oxide content in chromium tanned leather shaving is about 4.4%. Other authors have reported slightly lower chromium contents, namely between 2 and 4% [65,66]. Therefore, the main toxic compound found in the tanned leather waste is chromium which can be recovered by extraction and used again in the tanning process of leather. El Boushy et al. (1991) [67] utilized a method consisting of several washing steps (alternating alkaline-acid wash) of waste leather in order to decrease the chromium content. At the end the authors obtained a material rich in hide protein (74.9%), with a fibrous texture consisting mainly of collagen and having a digestibility of 98%. The chromium content was reported to be 0.2% [67]. A recent method consists of two steps: leaching with H_2_SO_4_ and ion exchange step using cation exchange resins, allowed to reduce the Cr (III) content at ppm level (14 ppm) [68]. However, this same process of leather stabilization will generate problems at the moment of waste leather disposal.

It has been evidenced that initially the cow hide has about 60–70% water, 30–35% proteins, 0.5–2% lipids and 0.35–0.5% mineral compounds. The tanning process determinates a decrease in the water content, which gives in the end a content of about 70% proteins (mostly collagen and small amounts of elastin) [69,70].

**Figure 3:**
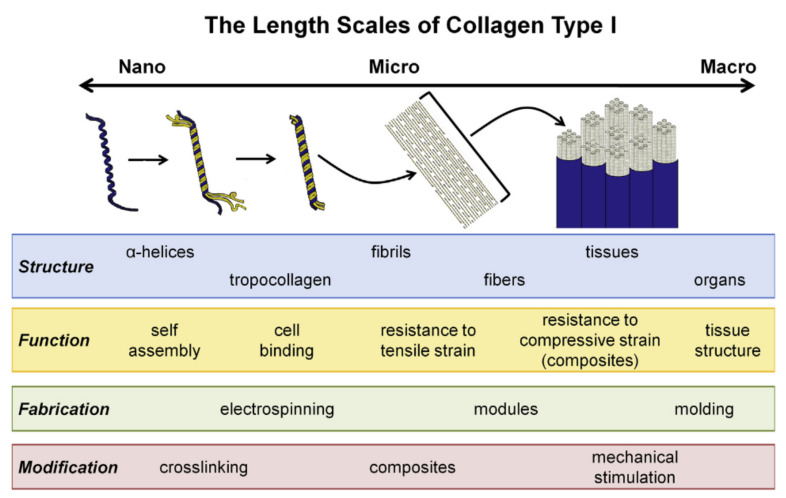
Representation of collagen type I structure at different scales [72].

The main protein that is encountered in hide wastes is the collagen that has 28 different types that can vary in abundance, distribution and functionality within a tissue; the most abundant is collagen type I that can form up to 90% of the connective matrix. A collagen molecule consists of three polypeptide chains assembled into a triple helix structure and a repeating amino acid sequence is responsible for the helical arrangement. [71]. Walters and Stegemann (2014) [72], described the collagen complex structure from the nano to macroscale emphasizing the role of collagen at each scale (Figure 3).

Two of the three polypeptide chains are identical, the third has a distinct sequence of amino acids. The α-chains are composed of repeating sequences of three amino acids that have a glycine at every third interval. Glycine amino acid allows the rotational freedom that leads to the helical structure. Other amino acids are responsible for stability, rigidity, biochemical and physical characteristics of collagen [72,73].

## 4. Preparation of Polymer Based Smart Fertilizer

A good fertilizer releases the nutrients in time and at the same time is biodegradable. The release of nutrients is based on the concentration gradient that exists between soil and fertilizer matrix. The fertilizer biodegradability allows an advanced release of the nutrients once the surface nutrients are consumed and at the same time provides the carbon necessary for the plant to grow.

**Figure 4:**
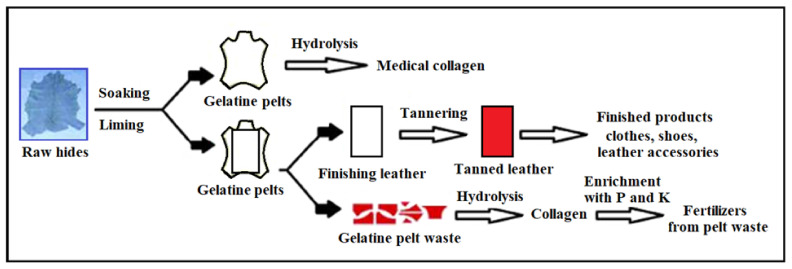
Differences in the preparation of medical collagen and agricultural collagen, readapted from [80].

Hydrogels are one type of polymer materials that have the advantage of absorbing and retaining high quantities of water, while they do not dissolve in contact with the water. This ability is given by the numerous functional hydrophilic groups (carboxylic acids, alcohols, amides and amines) attached to the polymeric chain, while the resistance to water dissolution is the result of cross-linked chains forming a three-dimensional network [74]. The high quantity of water retained, facilitates the diffusion of the nutrients through the polymer structure. Different types of hydrogels can be synthesized depending on the protein that is employed: collagen, gelatine, fibrin, silk, elastin, keratin [75]. Composite materials synthesized by hide waste hydrolysis are nontoxic compounds that have two major areas of utilization (Figure 4): (a) in medical applications and cosmetic products [75,76,77,78,79] and (b) in agriculture as fertilizers or as additive for animal feed [22,67,80,81]. In recent years, the use of collagen recovered from leather waste as food additive for animals feeding has been forbidden in the European Union [80]. The difference between the two major utilizations is that for the medical use the entire gelatine pelt is used while in the leather industry the gelatine pelts are processed (cutting, tanning) to obtain the final product and the waste is reused to extract the collagen.

To produce the biofertilizer, the first step is to recover the collagen from the hide waste. This is made by extraction via the hydrolysis process. A partial hydrolysis process gives rise to gelatine whilst a more advanced hydrolysis generates the collagen hydrolysate, a liquid that contains low molecular weight peptides [82,83]. The collagen hydrolysate is further modified by chemical cross-linking which consists in reactions between collagen’s reactive groups and the functional groups of a water-soluble copolymer. This step is necessary in order to overcome the disadvantages related to the liquid state of collagen hydrolysate which limits its utilization as fertilizer due to the odour, risk of microbial development and difficulty to be applied on the soils [84].

Masilamani et al. (2016) [85] presented a method for the extraction of collagen from trimming waste using acetic or propionic acid. Both acetic acid and propionic acid were effective in the extraction of collagen from trimming waste but propionic acid gives relatively higher amount of collagen extracted.

**Figure 5:**
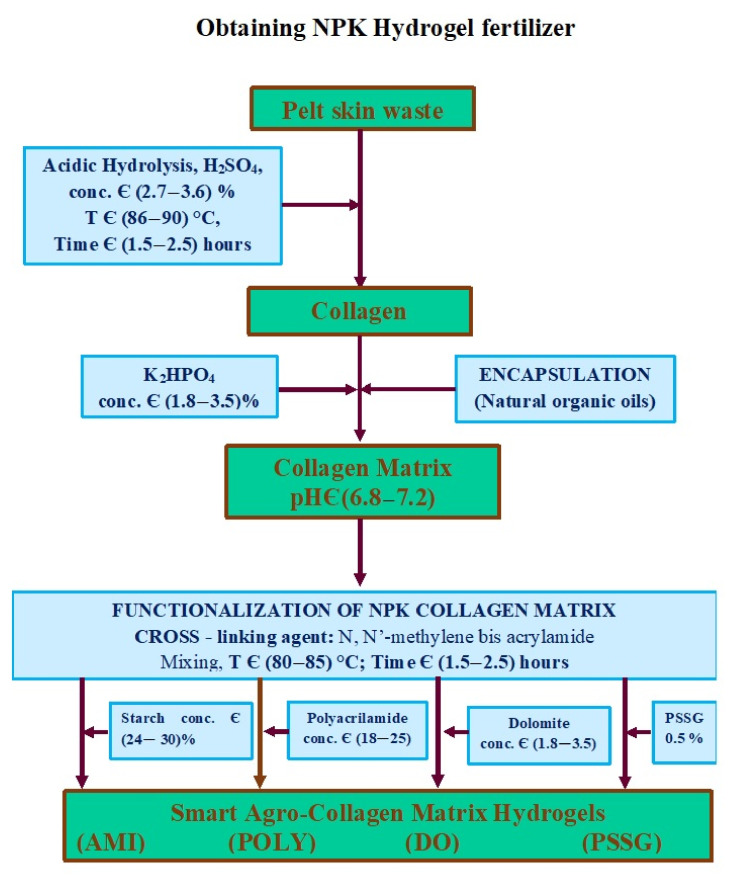
Technology scheme for obtaining smart-fertilizers by using acid hydrolysis, readapted from [80] (CH—collagen hydrolysate, Ref—CH—collagen hydrolysate with nutrients encapsulated as reference sample, PSSG—Ref—CH functionalized with P(SSNa—co—GMAx) copolymer, POLY—Ref—CH functionalized with polyacrylamide, AMI—Ref—CH functionalized with starch, AMI—Ref—CH functionalized with dolomite).

The steps of a smart fertilizer synthesizing starting from leather wastes, are depicted in Figure 5. In the first stage, the collagen matrix is obtained by hydrolysing the hide waste. The hydrolysis can be either using base [86] or acid chemicals [84]. The collagen hydrolysate is a liquid that contrary to medical applications, cannot be used as a fertilizer in this form. Therefore, a stabilization step is necessary by cross-linking with water soluble polymers. The resulted copolymers will incorporate more easily the nutrients, and release them later according to the plants’ needs.

**Figure 6:**
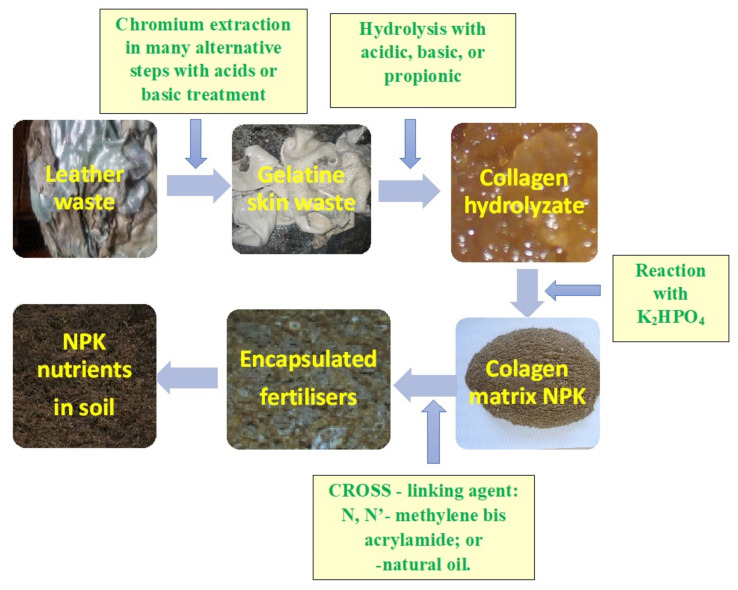
From waste leather to bio-polymer fertilizer—Intermediary products [87].

Figure 6 shows images of different intermediary products obtained during the synthesis of a smart fertilizer starting from wet blue wastes (resulted by leather tanning with chromium salt) [87].

Tzoumani et al. (2019) [84] selected poly (sodium 4-styrenesulfonate-co-glycidyl methacrylate) (P(SSNa-co-GMAx)) as water -soluble copolymer, because the behaviour of a charged polyelectrolyte combined with the reactive epoxy groups will be used in the cross-linking process. Different ratios of monomers have been used in the preparation of copolymers named P(SSNa-co-GMAx). The collagen hydrolysate was modified with P(SSNa-co-GMAx) or starch. To confirm the cross-linking between collagen hydrolysate and the epoxy groups, ATR-FTIR analysis was used. The authors have compared the variation of the release degree for oxidable compounds in water, in time, and found a controlled release in the case of enriched collagen functionalized with synthetic polymer and starch, compared to the un-functionalized enriched collagen (functionalization of collagen results in slowing their release capacity).

Hu et al. (2021) [16] described the preparation of a new fertilizer using leather waste as source of collagen, having the abilities of nutrient controlled release and heavy metal adsorption. The ability of heavy metal removal was evaluated under different environmental parameters (pH, coexisting ions), to demonstrate the potential application of this fertilizer as a green and sustainable agrochemical. The hydrogel was obtained by hydrolysis of leather waste, using KOH solution, at 60 °C under stirring. The protein hydrolysate is functionalized by adding a mixture of neutralized acrylic acid, ammonized maleic anhydride, N, N-methylene-bis-acrylamide, ammonium persulfate, and sodium bisulphite, at the weight ratio of 1000:100:0.55:2.2:1.1, at 60 °C. The product was swollen, washed, filtered and dewatered, in excess ethanol. Afterward, tests to evaluate the swelling characteristics, nutrient release ratios and biodegradation, were made.

In Table 1 and Table 2 a comparison among the different characteristics of the hydrogel-based fertilizers is presented. Higher pore diameter values determinate higher surface area to volume ratio which produces a fertilizer with enhanced swelling rate and biodegradability [16,81].

Some researchers combined the use of wet blue leather, as a source of nitrogen, with other materials (poultry bone meal and water hyacinth ash, as a source of phosphorous, and potassium, respectively) for the production of N-P-K (Nitrogen-Phosphorous-Potassium) enriched organic fertilizer [88]. The chromium was extracted from wet blue leather by basic hydrolysis, followed by acidic hydrolysis. The collagenic material was further mixed with the poultry bone meal, and potassium enriched water hyacinth ash, the resulted organic N-P-K based manure was then tested as a nutrient source for Catharanthus roseus (Madagascar Periwinkle). Results were compared with plant growth on soils without fertilizers, and on soils containing a conventional chemical fertilizer. It was found that the release of nutrients was controlled for the polymer-based fertilizer with a sustained plant growth over time, while the chemical fertilizer dissolved faster in the soil moisture and gave extra plant growth in the initial stage, but slower afterwards. In a more recent study, two organic ingredients, namely chromium-free collagen of wet blue leather (WBL) waste-as nitrogen source, and potato peel biochar-as potassium-phosphorus source, were used for the synthesis of NPK rich bio-fertilizer [89]. The chromium was extracted from wet blue leather by treatment with H_3_PO_4_ [90,91]; the so treated wet blue leather waste was washed with distilled water, and tested for residual chromium, both in the washing solution, and WBL, using atomic absorption spectrophotometer. The degree of chromium removal at the final washing step was of 90.38%. The potato peel biochar was washed with water to remove the residual dust, dried at 80 °C for humidity decrease, crushed and then carbonized in an electric furnace at 450 °C for 1 h. The two resulted materials WBL (as a nitrogen source) and potato peel biochar (as potassium and phosphorus source) were mixed in the ratio of 1:2.5. The mixture was dried at 30 °C for 24 h, and crushed, to obtain the bio-organic NPK fertilizer powder, which is easy to spread on the agricultural fields. The final product was checked by analytical characterization by SEM, EDS and FT-IR, and the final composition was: nitrogen 13.10%, phosphorus 2.41%, potassium 20.20% and magnesium 1.16%, carbon 33.74% and chromium 0.23%, indicating that all major nutrients are present as required by any commercial fertilizer. The biofertilizer was compared with a chemical fertilizer regarding their nutrients release in time: the chemical fertilizer released the nutrients more rapidly in time, causing a faster growth during the initial stage of the plant growth, whereas bio-organic NPK fertilizer loses nutrients in a controlled way, and determinates the plant growth uniformly in time. 

The influence of chromium was also discussed. The analysis of the soil composition before using the biofertilizer, indicated a concentration of 0.055 mg Cr/kg soil. The chromium content of the bio-organic fertilizer (0.71 mg Cr/kg) increased the soil chromium content to 0.765 mg Cr/kg soil, which is well below the maximum allowable limit of chromium in soils, which as recommended by WHO is 100 mg Cr/kg [92]. However, the authors proposed an advanced washing of the WBL with H_3_PO_4_, in order to further decrease the chromium content of the biofertilizer. 

Constantinescu et al. (2015) [93], presented the development of biocomposite fertilizers and their application in agriculture for plant growth (namely soybean crop), and remediation of soil content in required nutrients. For this purpose, the authors have used untanned waste provided by a local leather processing company, and the fertilizer was synthesized by alkaline hydrolysis of raw hide leather. Dipotassium phosphate was added, to improve the nutritional characteristics as regarding K and P. Results obtained on soils treated with this biofertilizer were compared to untreated soils, and showed that application of biofertilizer stimulated the plant growth, and the production increase. 

Zainescu et al. (2018) [94], synthesized a hydrogel based on collagen hydrolysate cross-linked with acrylamide synthetic polymer. Acrylamide was chosen because it offers several advantages: it is chemically inert, transparent and stable in a wide range of pH and temperature. The presence of cross-linking between collagen and polyacrylamide in the molecular structure of hydrogel was confirmed by optical microscopy and IR analysis.

Collagen recovered from wet blue leather wastes was used as adsorbent for K and P, in order to obtain an NPK-fertilizer [95]. The adsorption of P and K takes place in a multilayer at the surface of the adsorbent, and the process was very well described by Freundlich models. The resulted fertilizer was applied as a source of nutrients for promoting the growth of rice plants with promising results.

## 5. Biodegradation of Polymers Extracted from Hide and Leather Waste

The collagen is a natural polymer that by itself is enzymatically degradable [96]. Soil humidity and temperature are influencing factors that stimulate the biodegradation of hydrogels, under the influence of proteolytic bacteria [94]. The biodegradation time of hydrogels vary from one up to six months [80,97,98]. Generally, the enzymatic biodegradation takes place through the action of enzymes and/or chemical deterioration associated with living organisms.

Biodegradability depends on the polymer chemical structure and the environmental degrading conditions (pH, water availability, temperature, light) [99].

**Figure 7:**
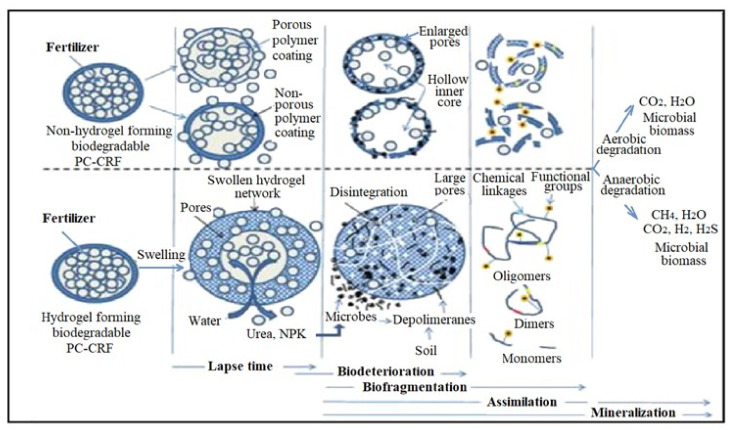
Biodegradation in soils of hydrogel compared with non-hydrogel polymers [100].

The biodegradation mechanism of polymer-coated controlled-release PC-CRT fertilizers in soils involves several steps (Figure 7) [100]:

(1) swelling: the ionic functional groups like hydroxyl, carboxyl, and amino can form the hydrogen bounds with water and more easily swell resulting in a porous network. This behaviour is specific only for hydrogels that have more functional ionic groups. The swollen porous structure increases the pore size, allowing the release of incorporated fertilizers such as urea, phosphates, etc.
(2) biodeterioration: polymer fragmentation into lower molecular mass species in abiotic reactions (oxidation, photodegradation, hydrolysis).
(3) biofragmentation: the polymer is fragmentated in biotic reactions, i.e., hydrolysis of macromolecules in oligomer, dimer or monomer, the mediators being microorganisms.
(4) assimilation: the monomer can be absorbed by the microorganisms and degraded for example by deamination or decarboxylation, resulting ammonia or nitrate, acids and alcohols etc.
(5) mineralization: is the process of degradation of organic compounds in aerobic and anaerobic conditions to mineral compounds (nitrate, carbon dioxide, hydrogen, methane).

In leather waste, the collagen is crosslinked with the tanning agent, which gives stability to biodegradation. In order to assess the suitability of using leather waste as fertilizers, and to evaluate their behaviour and potentially adverse environmental effects, studies of leather biodegradability have been conducted. Stefan et al. (2012) [70], made an experimental study on the identification of microorganisms that are suitable to be used in the improvement of waste leather biodegradation. Their investigation consisted in the isolation, selection and characterization of microorganisms that produce the extracellular protease and lipase. The inoculum of microorganisms was taken from an old waste storage dump leather. The Bacillus species showed higher extracellular proteolytic and lipolytic activity, the maximum production being obtained after 48 h. 

Because of the environmental issues related to the use of chromium-based salts in the leather tanning process, in recent years, methods employing vegetal materials as tanning agents have been developed [101,102,103,104,105]. A comparative study of the biodegradation in aqueous solution in aerobic condition of leather wastes, generated in the tanning process using chromium or vegetal compounds, for a period of 100 days, was made by Stefan et al. (2011) [106]. To reproduce the effect of nutrients in fertilizers, a mixture of nutritive salts containing K, N, P, Na, Ca was added, and the leather was used as carbon source for the crop growth, while as inoculum, the liquid extract of compost generated from leather waste disposal was used. Through this system, air free of carbon dioxide was bubbled at a flow rate between 1.5 ÷ 2.5 L/s, at constant temperature 20 ± 0.5 °C. Several parameters were monitored, in order to account for leather biodegradation process: carbon dioxide resulted, respectively pH, conductivity, TOC (total organic carbon) and TON (total organic nitrogen) of the liquid phase.

The biodegradability of untanned, chrome tanned and vegetal tanned leather under anaerobic conditions was investigated, and the effect of untanning on the leather biodegradability was reported [107]. As inoculum anaerobic microorganisms isolated from anaerobic sludge were used, generated by wastewater treatment plant employed for the treatment of tannery wastewater, and also from the sludge obtained from the sewage treatment plant treating domestic wastewater. The results showed that biodegradation of chrome tanned leather waste is possible using anaerobic sludge, and that in certain conditions, the degradation degree is higher than for the vegetable tanned leather waste (when the untanning process is introduced before the biodegradation process, the resulted material is less stable).

Zainescu et al. (2018) [94], studied the biodegradability of the collagen hydrogel with encapsulated nutrients by two methods: in soil, by measuring the weight loss, and in aerobic conditions in aqueous medium (according to SR EN ISO 14852/2005). The second method is recommended by the authors for evaluating the hydrogel biodegradability due to its accuracy.

Although not so high as the activated sewage sludge, soil has still an important biological activity, being rich in species that could be a source of inoculum. Stefan et al. (2020) [80] performed biodegradability tests for several fertilizers, in water in aerobic conditions, and in composting conditions. The experiments realized in composting conditions aim to study the fertilizer biodegradability in conditions similar to that encountered in agricultural environment. The tests were performed according to the standard procedures respectively SR EN ISO 14852/2005, for determination of the aerobic biodegradability in aqueous medium of plastic materials, and SR EN ISO 14855-1/2008, for determination of the final aerobic biodegradability in composting-controlled conditions. Both standards evaluate biodegradability based on the measuring of CO_2_ quantity released during the polymer consumption. Experiments evidenced the existence of four regions corresponding to the steps of biodegradation process:

(1) stagnant zone: 0 ÷ 6 days, the biodegradation process is initiated and the process rate is small.
(2) acceleration zone: 2 ÷ 50 days, the biodegradability is linearly increasing.
(3) slowing zone: 14 ÷ 56 days, the biodegradability rate is decreasing.
(4) stationary zone: 41 ÷ 75 days, biodegradation reaches its maximum degree and the biodegradability rate goes to zero.

The results indicated that the biodegradability degree is higher for the biopolymer-based products, and smaller in the case of compounds functionalized with synthetic polymers (polyacrylamide and P(SSNa—co—GMAx) copolymer, noted PSSG). As regarding the environment, biodegradation is slower in composting medium, than in water medium. The evolution of biodegradability in time, in water and in composting conditions, for the studied fertilizers, compared with collagen hydrolysate is shown in Table 3. As expected, the collagen hydrolysate (CH and Ref CH) has the highest biodegradability, followed by collagen functionalized with starch while the functionalized fertilizers with synthetic polymers have the lowest biodegradability (POLY and PSSG). 

In literature it is reported that 15–30% of the fertilizers remained unreleased from PC-CRFs due to the concentration gradient difference across the polymer coatings [108]. It can be seen that the amount of fertilizers remained unreleased in the case of gelatine-based fertilizers functionalized with natural polymers is in the range of 20–25% in time, and for those functionalized with synthetic polymers in the range of 32–34%.

There have been numerous studies performed on starch-based hydrogels functionalized with natural and synthetic polymers for example: chitosan, ethyl cellulose, polyacrylic acid, clay, lignin and polyurethane, polyvinyl alcohol, polybutylene succinates and other variants [19,108,109,110,111,112].

The unreleased amounts of fertiliser starch-based hydrogels functionalized with natural and synthetic polymers varied between 10–25%. Fertilizers obtained from both natural collagen-based and starch-based polymers release fertilizers more easily than synthetic ones. The degree of biodegradation is also higher in the case of fertilizers obtained from natural polymers. Furthermore, the degradation compounds obtained from fertilizers obtained from natural polymers are less toxic [100].

## 6. Release Mechanism of Nutrients

For the release mechanism of the nutrients, several stages were proposed: in a first step, organic and inorganic matter having high solubility (e.g., peptides with short chains, soluble polymers (starch), amino acids, glucides (mono- and disaccharides), nitrate salts, acids) is released. Afterwards, the release of oxidable compounds decreased for all fertilizers corresponding to organic compounds with low solubility (proteins) [80,84].

Du et al. (2006) [97], studied the influence of different parameters on the release of nutrients for two polymer coated compound fertilizers having the same core composition but different ratio in the nutrients of the coating. It was found that the nutrients release from CRF was mainly controlled by the diffusion mechanism. In this case, among the parameters that influence the diffusion, the temperature and coating thickness had an important role: lower temperature and thicker membrane determinate a lower diffusion coefficient of coated membrane, which slowed the nutrients release rate. Furthermore, nutrients release rate was different, depending on the diffusion medium, the fastest release rate was in water, then water saturated sand, and the last was in sand at field capacity. Nutrients release profile over 70 days indicate three steps: lag period, linear stage and decay period. The lag period of P was significantly longer than of other nutrients indicating that there is a strong bond between P and the core composition.

### 6.1. Hydrogel Modelling—Kinetic Modelling of Nutrients Release

There are few studies dedicated to modelling of hydrogel materials regarding the release of different chemicals. The first model used for fitting the experimental data of substance release from porous hydrophilic polymers is Korsmeyer-Peppas model [113,114,115,116]. The Nernst -Planck equation, accounting for the fluxes of mobile ions in the hydrogel structure and in external diffusion layer has been used by Pareek et al. (2017) [117] and Goh et al. (2017) [118]. Assuming that the hydrogel pores are narrow enough so that the diffusion dominates the transport across the hydrogel and that the nutrients are uniformly dispersed throughout the matrix, unsteady-state nutrient diffusion in a one-dimensional direction can be described using Fick’s second law of diffusion [119]. Mass conservation balance is expressed in a mathematical equation that accounts for diffusion of species inside and outside hydrogels, electrostatic interactions, hydrophobic associations, cleavable covalent linkage and degradation [80]. An empirical equation developed by Peppas et al. (2000) [120] assumes a time-dependent power law function for the released quantity of nutrient:

R_f_ = k t*^n^*

where R_f_ represents the release factor,

k—kinetic constant dependent on material

*n*—exponent depending on type of transport, hydrogel geometry and polymer polydispersity; values of *n* close to 0.5 indicate that the controlling step in the transport mechanism is the diffusion while for *n* values close to 1, the surface deterioration is the controlling step [80].

The kinetic parameters, as reported by Stefan et al. (2020) [80], are given in Table 4, being calculated from experimental data for the leaching degree of oxidable compounds for the tested fertilizers, during almost one month (27 days). The leaching test consisted in the determination of chemical oxygen demand with KMnO_4_ (CODMn).

The values of the exponent *n* are above 0.5, indicating that the transport mechanism is controlled by several steps. The kinetic parameters were recalculated using a logarithmic form, and two linear regions were identified corresponding to: (1) the initial step days 3–10, *n* > 1, (2) the second step *n* is around 0.5. In the first zone, the controlling step is attributed to surface deterioration by KMnO_4_, while in the second zone, the controlling step is the diffusion of oxidation compounds.

### 6.2. Hydrogel Modelling—Swelling Process

The model mentioned above considers only the nutrients transport across the matrix. More complex models include also the water transport and the hydrogel swelling/shrinkage [121]. The hydrogel swelling depends on a series of parameters such as temperature, pH, solvent and hydrogel structure, because the swelling process is thermodynamically controlled by hydrogel-solvent interactions [15]. The equilibrium state for the swelling process of the smart hydrogel in solvent is reached when the solvent inside the hydrogels is in thermodynamic equilibrium with that outside [15,122].

Hydrogel swelling ratio is defined as follows:

S.R. = (m_hydrated_ − m_dehydrated_)/m_dehydrated_

where S.R. is the swelling ration and m is the mass of either hydrated or dehydrated hydrogel.

Activity coefficients for species in bulk volume or in hydrogel phase were calculated using either UNIQUAC or NRTL models [123,124,125]. Recent papers have reported, based on experimental-modelling data comparisons, that NRTL model is more suitable to be used, as UNIQUAC model fails to accurately account for liquid phase-hydrogel interaction in cross linked polymers [15].

Sheth et al. (2019) [126] developed an 1D (one-dimensional) computational model for the diffusion and swelling, that accounts for time-varying of diffusivity and geometry to predict profiles of substances released from degradable hydrogels. Time snapshots of diffusivity and hydrogel geometry data measured experimentally were used as inputs in the computational model, which predicted the components profiles.

A three-phase complex model (a solid matrix, the hydrogel and the liquid solvent) was developed by Sauerwein and Steeb (2020), model that has been validated by experimental data obtained for hydrogel swelling in different solvents [122]. The governing equations of the model for mass, momentum and electrostatic charges, were written assuming isothermal conditions [127]. However, their model has to be adapted when used for the fertilizers case, because the fertilizers do not include the matrix phase.

Three steps are influencing the swelling behaviour of a hydrogel: the local polymer and ionic liquid phase concentrations, the elasticity of the polymer network (given by the crosslinking degree), and the behaviour of the ions at the hydrogel-liquid phase interface governed by Donnan equilibrium [128]. As regarding the swelling kinetics, this is mainly limited by the diffusion step [129]. However, work still needs to be done regarding the approximation of swelling/deswelling time, which are not equal.

## 7. Future Perspectives of the Production of Smart Fertilizers from Leather Waste

In order to identify the nutritional status and, consequently the needed fertilizer dose, it is necessary to know the initial state of soil fertility (defined by the agrochemical characteristics of the soil), the species and variety of plants to be cultivated, and the type of the ecological zone (i.e., if there are underground waters that are susceptible to be polluted by the levigated nutrients).

The choice among the different types of smart fertilizers obtained using collagen hydrolysate obtained by acidic, basic or enzymatic hydrolysis is made after the characterisation of soils. The soil composition regarding the initial content in nutrients (mainly N, P, K) and acidity must be known prior to the decision whether to use, for example, a P-enriched smart fertilizer, or a more acid, or a more basic one. During the synthesis steps, the ratio between the nutrients N, P, K and the pH of the final product can be adjusted to correspond to soil deficiencies and to the crop demand.

The use of enzymatic hydrolysis is highly recommended, when the final fertilizer is intended to be applied on soils with high salinity, and for which it is not recommended a further increase in the salt content.

As chromium containing components are dangerous for human health and for the environment, chromium content should be carefully checked when the fertilizer is synthesised by using tanned wastes as its content must be in agreement with the imposed regulations. This step will not add a high extra cost to the fertilizers final price, because the tanneries employing chromium in their processing are already recovering Cr containing compounds before waste disposal to landfilling.

## 8. Conclusions

The scope of this review was to highlight the potential of hide waste as a source of collagen-based biopolymers. The characteristics of hide leather wastes have been presented and, as well as the problems generated, if these wastes are left untreated. The methods for the synthesis of smart fertilizers have been detailed, together with the advantages generated by reducing the impact on the environment (by recovering resources from wastes and by offering a bio-fertilizer as an alternative to the chemical fertilizer).

The biggest advantage of this type of fertilizers is that the synthesis method can be easily adapted to the beneficiary demands, namely it can be adjusted to soil characteristics (pH and nutrients ratio), as well as to crop necessity in nutrients.

The main advantage that arises from replacing the conventional chemical fertilizers with bio-polymer containing fertilizers is related to the gradual release of nutrients over a longer period of time, which leads to increasing crop production and improving the quality of plants by controlled release of nutrients. On the contrary, in the case of chemically fertilizers, the release of nutrients is immediate, and if combined with the meteorological conditions (e.g., rains), it can lead to the nutrients’ leachability in the deeper layers of the soil and aquifer causing major pollution problems.

The smart fertilizers obtained by enzymatic hydrolysis of leather wastes are indicated for soils with high salinity. This ensures the reduction of stress caused by increasing further the soil salinity, or by applying treatments with phytosanitary products. This type of smart fertilizer improves the beneficial activity of microorganisms in the soil, and increases the permeability of cell membranes from the root system, favouring the nutrients absorption and retention.

In the case of alkaline soils (which are the worst soils for plants growth), collagen-based fertilizers act as a naturally chelating agent for micronutrients, favouring their accessibility to the plant.

The capacity to retain high quantities of water and to gradually release it reduces the irrigation frequency, and prevents water loss by evaporation.

The recovery and reuse of high quantities of waste generated by the leather industry by extracting a valuable raw material: the collagen, ensures the compliance of leather industry with more strict regulations related to waste disposal and in agreement with the principles of circular economy.

## Figures and Tables

**Table 1:** Comparison among different types of biopolymer-based fertilizers.

Hydrogel	Water Absorption Capacity	Cr (III) Adsorption Capacity
Biofertilizer (collagen-nitrogen and potassium) [16]	2208 g H_2_O/g	149.3 g Cr (III)/g
Biofertilizer (source P nutrient) Collagen-g-poly(acrylic acid-co-	2595 g H_2_O/g	Not tested
2-acrylamido-2-methyl-1-propane sulfonic acid)–iron(III) [81]		
Collagen-polyacrylic acid-co-		
2-acrylamido-2-methyl-1-propane sulfonic acid) [81]	3578 g H_2_O/g	Not tested

**Table 2:** Characteristics of hydrogels.

Hydrogel	Pore Diameter	Days of Controlled Nutrient Release
Biofertilizer (collagen-nitrogen and potassium) [16]	1.26–6.73 μm	More than 40
Biofertilizer (source P nutrient) Collagen-g-poly(acrylic acid-co-2-acrylamido-2-methyl-1-propane sulfonic acid)–iron(III) [81]	4–9 μm	More than 30

**Table 3:** Biodegradation degree evolution in time * [80].

Time, Days	Biodegradation Degree							
	CH	Ref CH		AMI		POLY		PSSG
	W	W	C	W	C	W	C	W
10	42	35	17	37	33	15	12	20
20	58	48	33	50	48	27	21	35
75	99	74	50	80	64	62	40	63

* CH—collagen hydrolysate, Ref-CH—collagen hydrolysate with nutrients encapsulated as reference sample, PSSG—Ref—CH functionalized with P(SSNa—co—GMAx) copolymer, POLY—Ref—CH functionalized with poly-acrylamide, AMI—Ref—CH functionalized with starch, W—water, C—composting conditions.

**Table 4:** Kinetic parameters values for compounds release * [80].

Fertilizer Type	k	*n*	R2
CH	0.1370	0.6693	0.9159
REF-CH	0.0480	0.9514	0.9240
AMI	0.0880	0.7995	0.9212
POLY	0.0560	0.9059	0.9212
PSSG	0.0710	0.8644	0.9097

* CH—collagen hydrolysate, Ref-CH—collagen hydrolysate with nutrients encapsulated as reference sample, PSSG—Ref—CH functionalized with P(SSNa—co—GMAx) copolymer, POLY—Ref—CH functionalized with poly-acrylamide, AMI—Ref—CH functionalized with starch.
